# Posttraumatic Fat Necrosis Presented as Cellulitis of the Leg

**DOI:** 10.1155/2012/672397

**Published:** 2012-09-10

**Authors:** Einat Haikin Herzberger, Shraga Aviner, Evgenia Cherniavsky

**Affiliations:** ^1^Department of Pediatrics, The Barzilai Medical Center, 2 Hahistadrut Street, Ashkelon 78278, Israel; ^2^Faculty of Health Sciences, Ben-Gurion University of the Negev, Beer-Sheva 84105, Israel; ^3^Department of Medical Imaging, The Barzilai Medical Center, Ashkelon 78278, Israel

## Abstract

Cellulitis, a diffuse inflammation of connective tissue with severe inflammation of dermal and subcutaneous layers of the skin, is a common lesion in children, usually responsive to systemic antibiotic therapy. However, an unusual course of healing or some nontypical features should call the treating physician to consider and investigate for other diagnoses that might prevent unnecessary treatment and alleviate parental stress. We present a case of posttraumatic fat necrosis, demonstrating some pitfalls in the process of diagnosis.

## 1. Introduction

Posttraumatic fat necrosis of the subcutaneous fat tissue can occur following a fall, blunt injury, surgery, and minor procedures such as injections [1]. It is more prevalent in women and usually appears on the shins, thighs, arms, breasts, and buttocks [2]. In children the most common site is the cheek following injury to the face. Usually a hematoma develops at the site of injury to be followed by a deeper induration [3]. It may appear as an incidental palpation of a lump, local depression, discoloration of the skin, or as a painful region [1]. As a history of injury is absent in many cases, the clinical picture may resemble a manifestation of a neoplastic disorder [1]. 

## 2. Case Presentation

A 9-year-old girl was admitted to the Emergency Department 4 days after a blunt trauma in her left leg. On admission the patient had no fever. On physical examination, there was an area of edema, warmth, erythema, and tenderness with indistinctive margins on the anteromedial aspect of the left leg.

The rest of the physical examination was normal except for obesity and her history was unremarkable. X-ray of the leg was normal. She was clinically diagnosed as having cellulitis and was discharged with oral amoxicillin/clavulonic acid. However, edema, warmth, erythema, and tenderness of the leg persisted for 2 more days, denoting no improvement and the patient was readmitted 2 days later to the Pediatrics Department. Oral antibiotic was replaced with intravenous therapy (amoxicillin/clavulanic acid 75 mg/kg/day). 

Laboratory findings were white blood cell count 13.6 × 10^3^/*μ*L (neutrophils 74.4%, lymphocytes 17.8%, monocytes 6.8%, eosinophils 0.8%), hemoglobin 13.0 g/dL, platelets 292 × 10^3^/*μ*L, C-reactive protein 94.1 mg/dL, erythrocyte sedimentation rate 67 mm at 1 hour, blood chemistry: normal. 

The antibiotic therapy was changed 4 days later to cloxacillin (100 mg/kg/day) and ampicillin (100 mg/kg/day) because of unsatisfying improvement.

Gradual improvement occurred over the next two days; edema, warmth and erythema became mild and the tenderness disappeared. The patient was discharged 6 days after her admission with oral cephalexin (35 mg/kg/day). 

**Figure 1:**
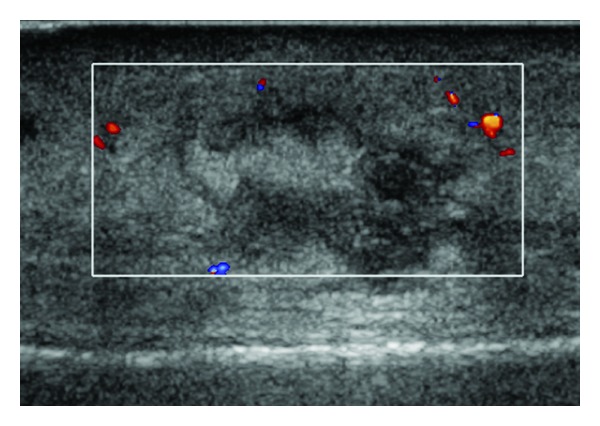
Ultrasound doppler 14 days after trauma. The investigation was targeted to the distal part of the shin. A complex process is seen in the subcutaneous fat with hyper- and hypoechoic regions and unclear boundaries without increased blood flow and without periosteal reaction.

Physical examination on a scheduled outpatient visit three days after discharge revealed erythema and induration of the skin of the left leg without tenderness or edema laboratory findings were within normal range. Ultrasound of the left shin demonstrated swelling of the soft tissue in the anteromedial aspect with a number of hypoechoic collections, the biggest one was 0.9 × 2.13 × 2 cm, margins were indistinctive with unclear fluid, blood supply was not increased, and the bones were intact, thus excluding the possibility of osteomyelitis (Figure 1). At this point the impression was of infection of deep tissues of the leg and antibiotic therapy was continued. On her next visit, three weeks on cephalexin, there were still erythema, stiffness, and induration of skin, less than what was seen on her last physical examination. Repeated ultrasound demonstrated swelling of the subcutaneous tissue without localized collection or abscess. The prolonged duration of clinical signs and the radiological findings prompted us to order a magnetic resonance imaging (MRI) study. 

**Figure 2:**
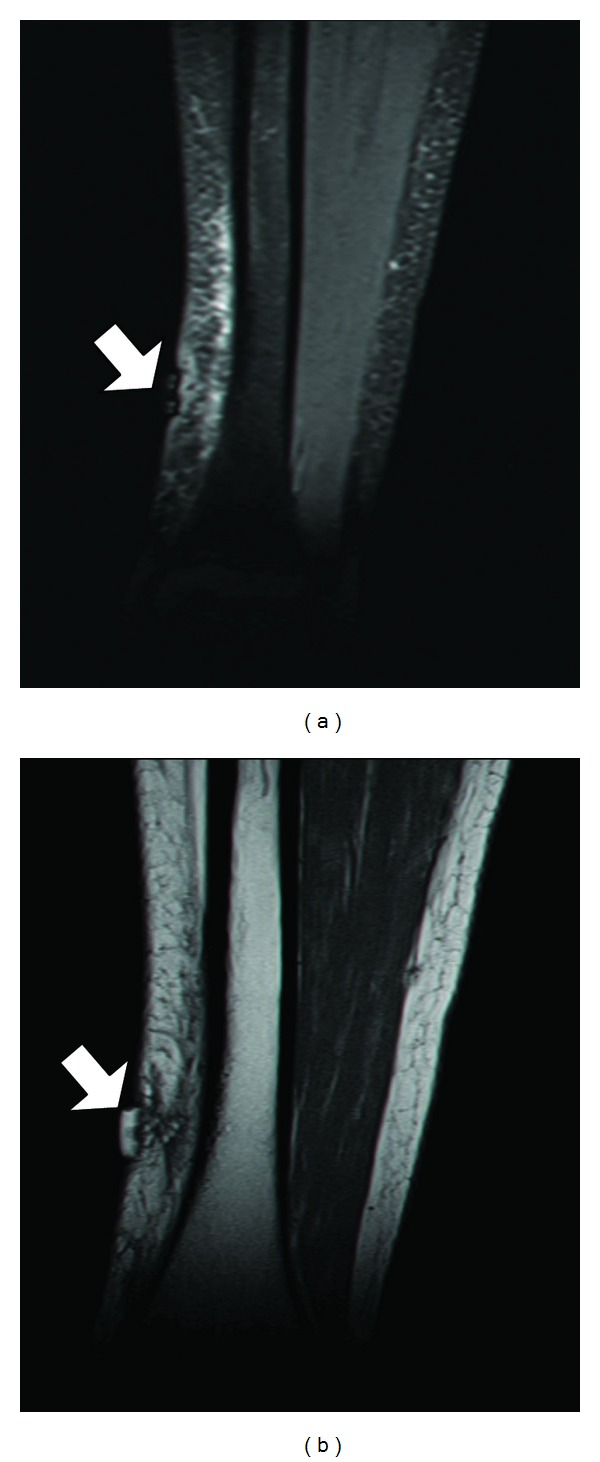
(a) MRI-T1 tirm and (b) MRI-T1. Coronal slices of the distal part of shin with cutaneous tag. MRI findings were edema in subcutaneous fat medial to tibial bone with thickening of subcutaneous septations and unclear boundaries, without involvement of bone cortex or marrow. T2-weighed images did not add information and are not shown.

Two months after admission, MRI demonstrated edema of the subcutaneous fat medially to the tibial bone with thickening of the subcutaneous septations and unclear boundaries. There was no involvement of bone cortex or marrow and no enhancement was evident (Figure 2). This picture is consistent with fat necrosis.

**Figure 3:**
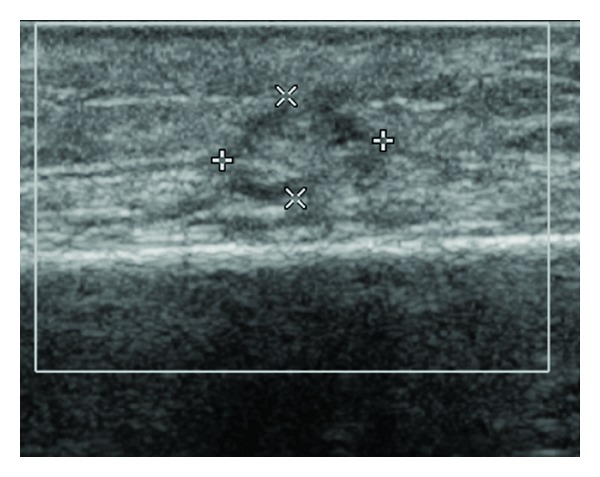
Ultrasound doppler 3 months after trauma. Resolution of the soft tissue edema: hyperechoic (fatty) lesion with hypoechoic halo with relatively clear boundaries. No periosteal reaction was noted.

Follow-up ultrasound 3 month after trauma showed resolution of soft tissue edema with organization of echogenic (fatty) lesion surround by hypoechoic lesion (Figure 3). 

## 3. Discussion

Posttraumatic fat necrosis is quite prevalent, especially in the pediatric age group where minor trauma is a normal and common event, making this population more prone to this condition [4]. Although this is a relatively common entity, there is low awareness to its existence. It has no serious medical consequences and does not require treatment other than symptomatic palliation [1]. However, it is important to be familiar with this entity which can mimic other conditions, and the reassuring statements concerning subcutaneous fat necrosis should not prevent further evaluation in an atypical clinical presentation. 

Most cases of posttraumatic fat necrosis appear as a palpable lump or as a local depression [1, 4]. Subcutaneous fat necrosis in children account for 13% of referrals for an MRI of softtissue mass [4]. In one case of posttraumatic subcutaneous fat necrosis appearing as a lump, the redness and ulceration appeared after frequent rubbing of the area and traumatizing the lesion [2]. Often the interval between the trauma and the initial observation of the lesion is prolonged [4], and a history of trauma may not be obtained, adding to the perplexing diagnosis.

The index case appeared with edema, warmth, erythema and tenderness—a clinical picture similar to cellulitis. Our patient had a prolonged duration of symptoms uncharacteristic of cellulitis. In a patient with cellulitis, a symptomatic improvement is expected within 24–48 hours of the beginning of therapy and a visible improvement within 72 hours. Persistence of symptoms or signs after the expected time prompts further evaluation and imaging of the affected area to rule out other possible diagnoses such as subcutaneous fat necrosis, tumors such as lymphoma, sarcoma, chloroma and early stages of necrotizing fasciitis and other rare infections. 

The appearance of our patient resembles that of earlier descriptions with gender distribution, typical location of lesion and recent history of trauma, and is different in that there is lack of a lump or depression at the site of the lesion. An alternative diagnosis that could explain these differences could be cellulitis with an extension to deeper layers, also known as infectious panniculitis. Primary infection can result from a pathogen introduced directly into the subcutaneous tissue through a penetration of the skin [5]. However, a diagnosis of infectious panniculitis in the pediatric age group is restricted almost exclusively to children who are immunocompromised and is rare even among this group [5]. Taking into account our patient's unremarkable prior medical history and the typical radiographic findings, this diagnosis is unlikely. Another explanation of this unusual clinical appearance is that this child appeared to be in the early evolving phase of the lesion, and a prolonged course might end up with a lump or depression of skin. This could be supported by the relatively short interval (4 days) from trauma to presentation compared to a longer interval of few weeks in cases described in literature [1, 4].
